# Supplementary material for: Net widening of Southern California beaches
Source: Nat Commun. 2026 Jan 29;17:1705. doi: 10.1038/s41467-026-68880-9 (PMC12909817; doi:10.1038/s41467-026-68880-9)
Supplement: Supplementary file 1 — Supplementary Information [file 41467_2026_68880_MOESM1_ESM.pdf]

Supplementary Information

Net Widening of Southern California Beaches

Jonathan A. Warrick[1*], Kilian Vos[2], Dan Buscombe[1,3], Andy Ritchie[1], Sean Vitousek[1], Teresa Hachey[4], and Brett Sanders[4]

[1]U.S. Geological Survey, 2885 Mission Street, Santa Cruz, CA 95060

[2]OHB Digital Services, Konrad-Zuse-Str. 8, 28359 Bremen, Germany

[3]Now at: Applied Coastal Research and Engineering, Washington State Department of Ecology, Olympia, WA 98513

[4]Department of Civil and Environmental Engineering, University of California, Irvine, Irvine, CA 92697

*Corresponding author (jwarrick@usgs.gov)

Contents:

Supplemental Figures S1-S13

Supplemental Tables S1-S2

References Cited

Supplemental Figures

Supp. Fig. S1. Integrated beach change for the northern, central, and southern regions of California.

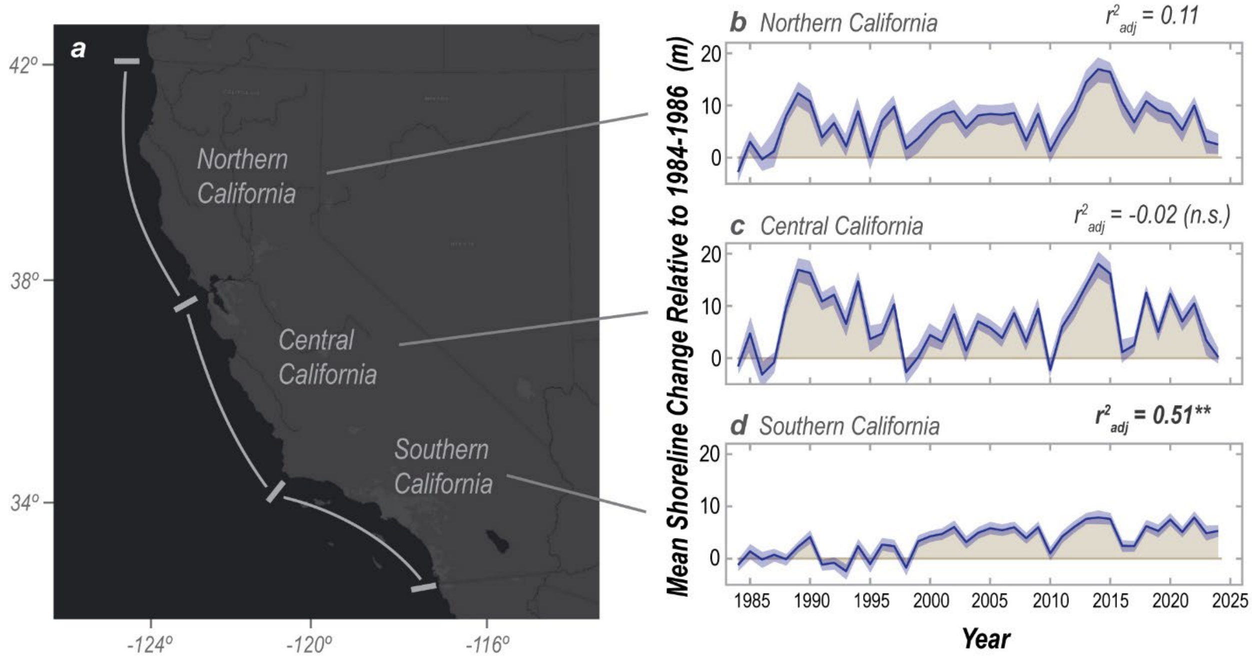

(a) Site map showing the three primary geographic regions of California that integrated shoreline position changes were calculated. (b-d) Integrated shoreline position changes averaged over the total beach length of northern, central, and southern California from the mean 1984-1986 value. Blue shading about the data represent 2-σ total uncertainty (see Materials and Methods). Tan shading represents the differences between annual values and the initial 1984-1986 mean value. For each region the adjusted correlation coefficient (r^2_{adj}) is provided for linear regression through the time series, which is denoted as highly significant ($p<0.001$) with bold text and stars, significant ($0.001<p<0.05$) with normal text, or not significant ($p>0.05$) with "n.s." A similar figure showing integrated beach area changes (in m^2) is provided in Figure 1 of the paper.

Supplemental Fig. S2. Beach changes in the Santa Barbara littoral cell of California.

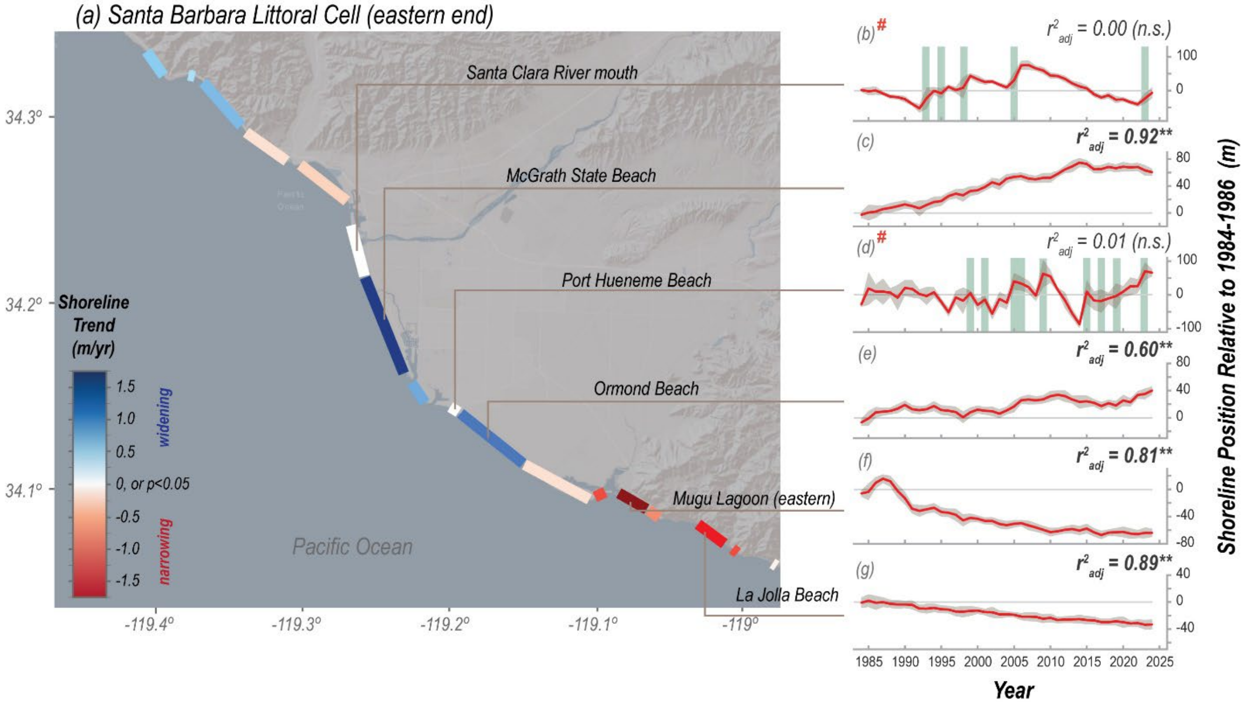

(a) Site map showing the eastern end of the Santa Barbara Littoral Cell (after Fig. 4a). (b) – (g) The integrated beach width changes for several of the beach segments of this cell. The shading about the lines represents 1-σ total uncertainty. For each region the adjusted correlation coefficient (r^2_{adj}) is provided for linear regression through each time series, which is denoted as highly significant ($p<0.001$) with bold text and stars, significant ($0.001<p<0.05$) with normal text, or not significant ($p>0.05$) with "n.s." Green bars in (b) – (g) represent sediment input events from river discharge, beach nourishment or dredging.

Supplemental Fig. S3. Beach changes in the Santa Monica littoral cell of California.

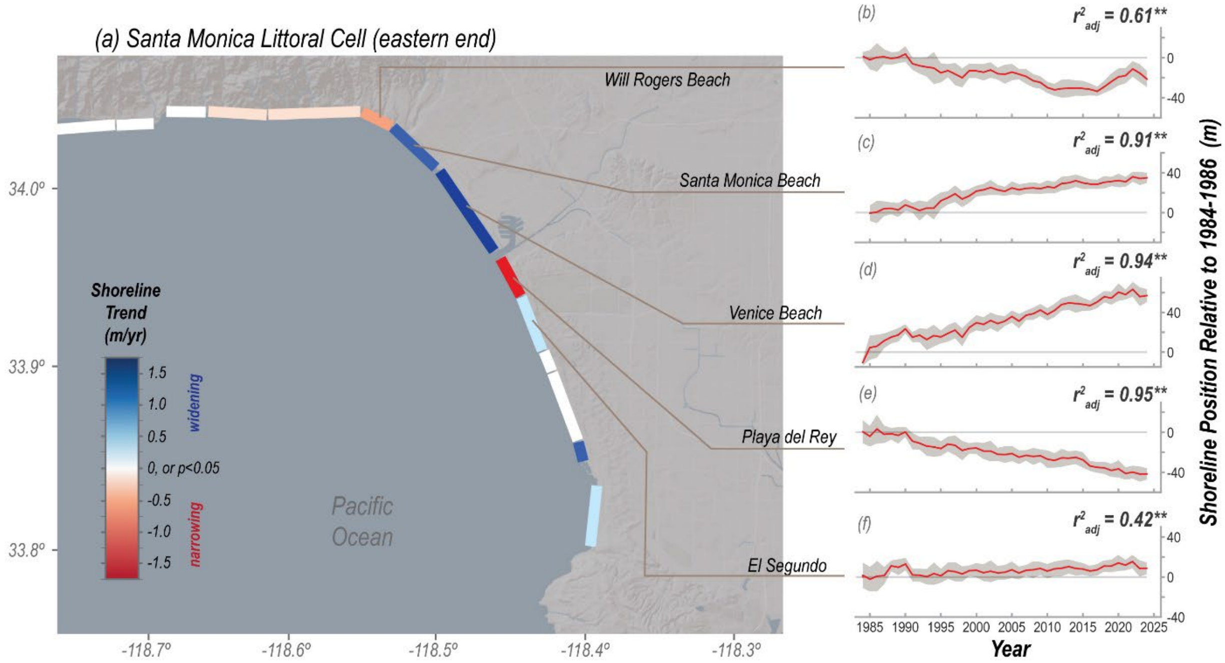

(a) Site map showing the eastern end of the Santa Monica Littoral Cell (after Fig. 4b). (b) – (f) The integrated beach width changes for several of the beach segments of this cell. The shading about the lines represents 1-σ total uncertainty. For each region the adjusted correlation coefficient (r^2_{adj}) is provided for linear regression through each time series, which is denoted as highly significant ($p<0.001$) with bold text and stars, significant ($0.001<p<0.05$) with normal text, or not significant ($p>0.05$) with "n.s."

Supplemental Fig. S4. Beach changes in the San Pedro littoral cell of California.

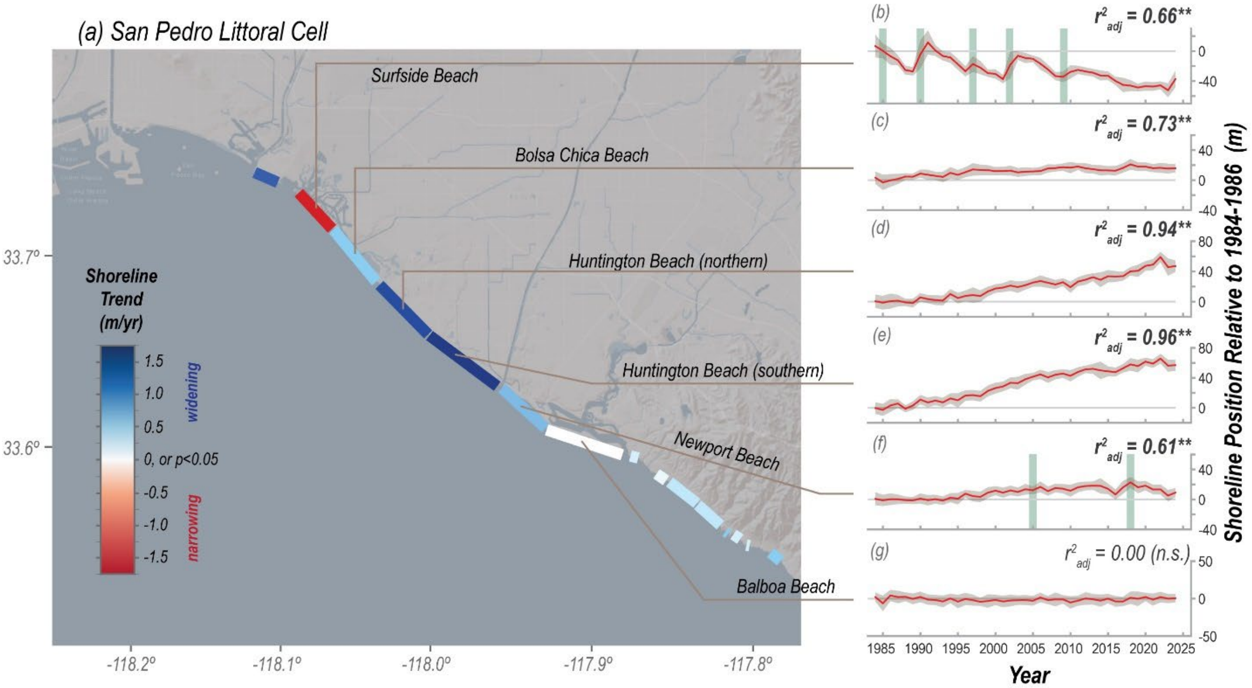

(a) Site map showing the San Pedro Littoral Cell (after Fig. 4c). (b) – (g) The integrated beach width changes for several of the beach segments of this cell. The shading about the lines represents 1-σ total uncertainty. For each region the adjusted correlation coefficient (r^2_{adj}) is provided for linear regression through each time series, which is denoted as highly significant ($p<0.001$) with bold text and stars, significant ($0.001<p<0.05$) with normal text, or not significant ($p>0.05$) with “n.s.” Green bars in (b) – (g) represent sediment input events from river discharge, beach nourishment or dredging.

Supplemental Fig. S5. Wave power hindcast data for southern California.

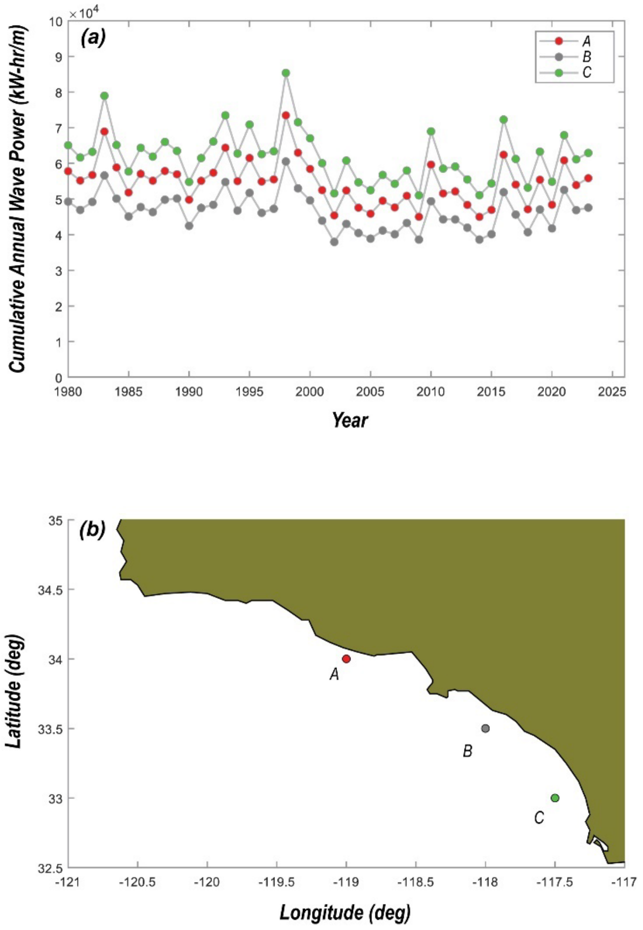

Annual values of the cumulative wave power from ERA5 hindcasts for southern California. (a) Annual time series of wave power for three ERA5 nodes that are nearest to the southern California littoral cells. Correlation (r^2) between these three sites exceed 0.95. (b) Locations of the ERA5 nodes for wave hindcasts.

Supplemental Fig. S6. Beach changes in the Zuma littoral cell of California.

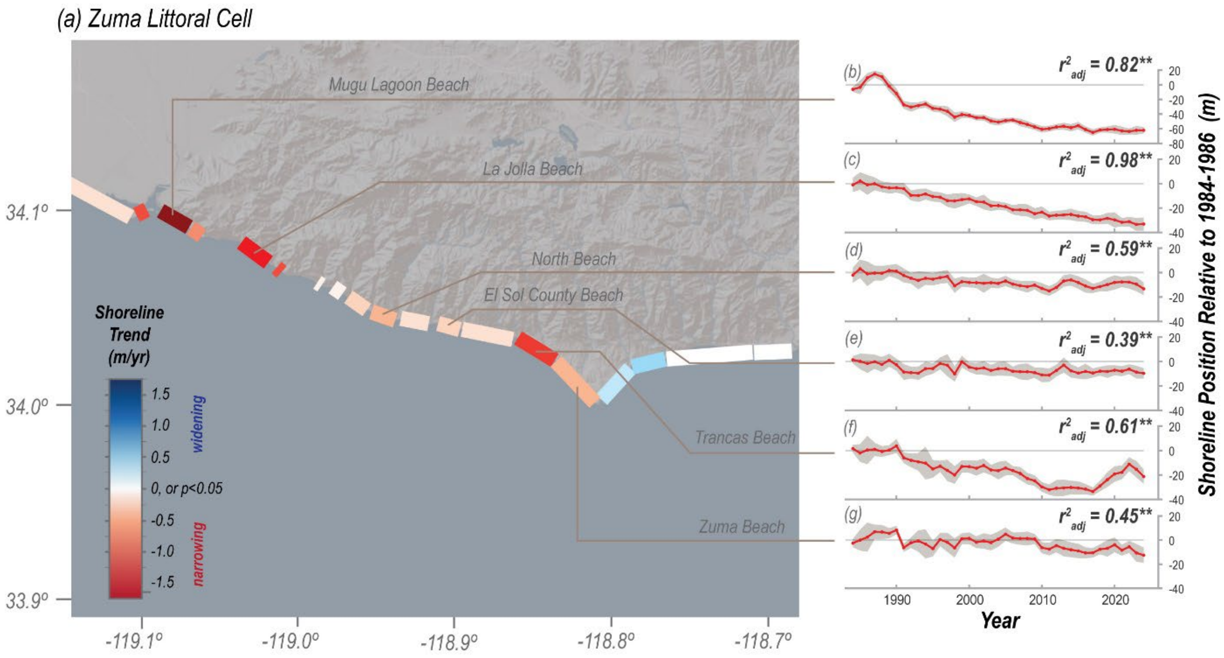

(a) Site map showing the Zuma Littoral Cell (after Fig. 5a). (b) – (g) The integrated beach width changes for several of the beach segments of this cell. The shading about the lines represents 1-σ total uncertainty. For each region the adjusted correlation coefficient (r^2_{adj}) is provided for linear regression through each time series, which is denoted as highly significant ($p<0.001$) with bold text and stars, significant ($0.001<p<0.05$) with normal text, or not significant ($p>0.05$) with “n.s.”

Supplemental Fig. S7. Beach changes in the Oceanside littoral cell of California.

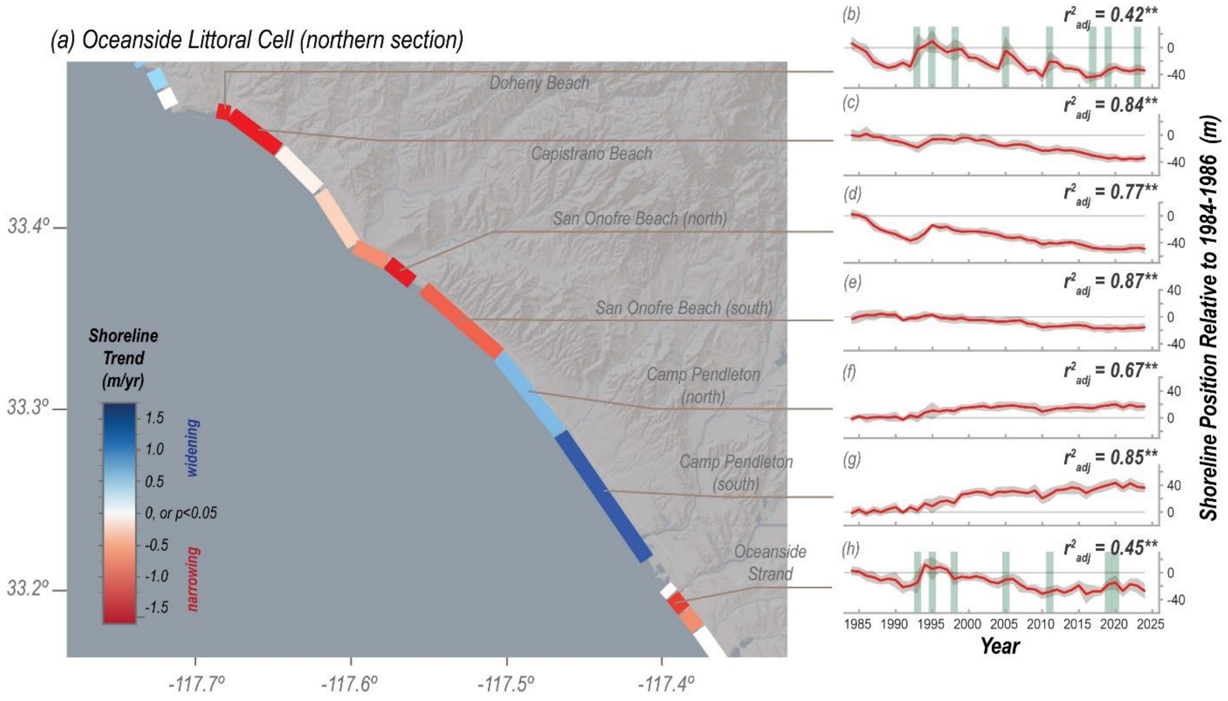

(a) Site map showing the northern portion of the Oceanside Littoral Cell (after Fig. 5b). (b) – (h) The integrated beach width changes for several of the beach segments of this cell. The shading about the lines represents 1-σ total uncertainty. For each region the adjusted correlation coefficient (r^2_{adj}) is provided for linear regression through each time series, which is denoted as highly significant ($p<0.001$) with bold text and stars, significant ($0.001<p<0.05$) with normal text, or not significant ($p>0.05$) with "n.s." Green bars in (b) and (h) represent sediment input events from river discharge as defined by peak annual river discharge in San Juan Creek and San Luis Rey River greater than 1000 m^3/s as measured at USGS gaging stations 11046530 and 11042000.

Supplemental Fig. S8. Decomposition method to compute annual average shoreline positions from CoastSat shoreline data.

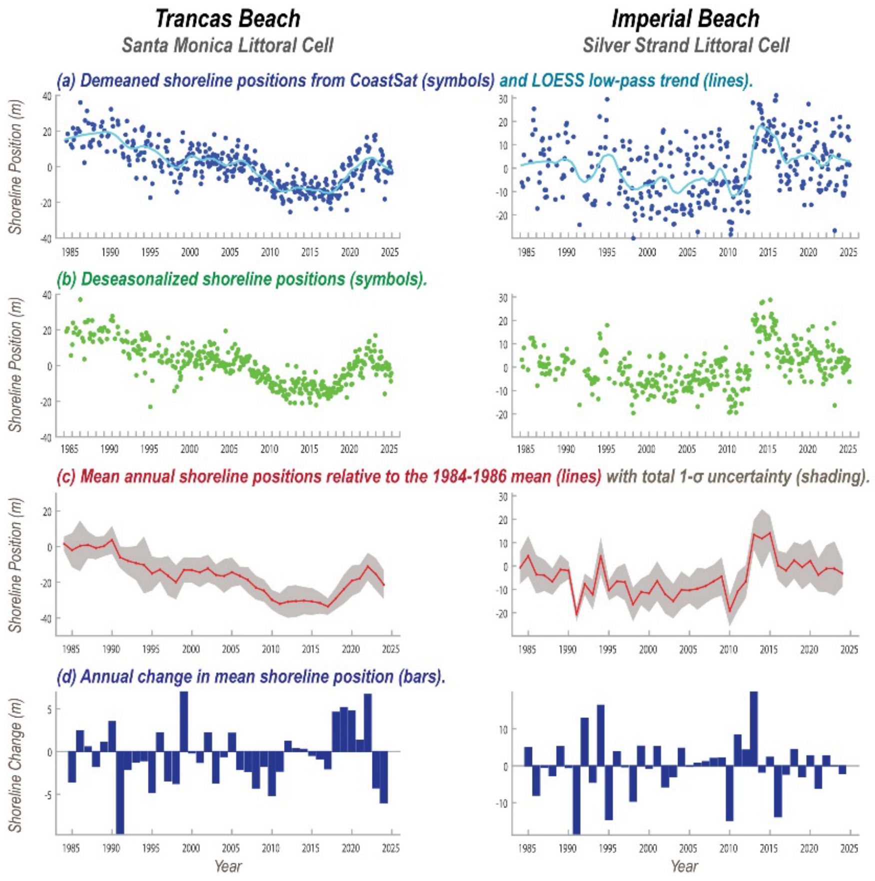

Presentation of the methods for computing mean annual shoreline positions for California beach segments following the Seasonal-Trend Decomposition with LOESS (STL) technique of Cleveland et al.[1] as implemented by Warrick et al.[2] (a) Demeaned shoreline positions for two beach segments (points) and LOESS low-pass fitted line following parameterization of Warrick et al.[2] (line). (b) Removal of the shoreline seasonality following the STL technique results in deseasonalized shoreline positions (points). (c) Mean annual shoreline positions and total 1-σ uncertainty. (d) Annual changes in mean shoreline position.

Supplemental Fig. S9. Monthly cloud-free satellite image counts during 1984-2024 for several example beach segments of California.

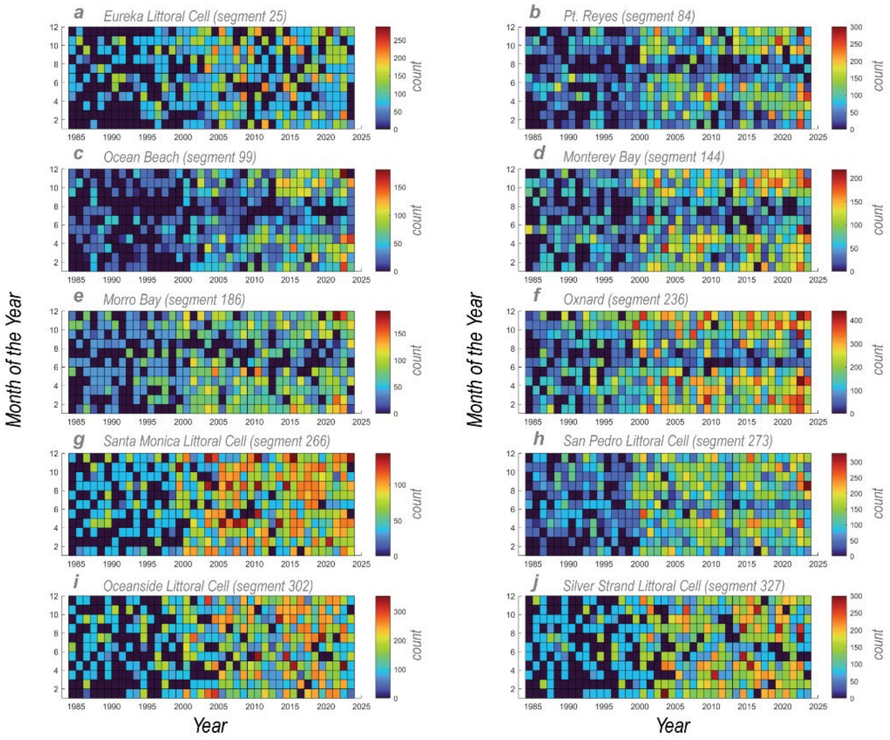

Cloud-free satellite imagery availability for ten beach segments of California showing monthly-to-multi-season data gaps. These data gaps are especially prevalent during 1984-1999 when only one satellite data source, Landsat 5, was available. Data availability is more consistent during 2000-2024 when two or more satellites were providing data.

Supplemental Fig. S10. Shoreline positions from field surveys of southern California beaches by Ludka et al.[3]

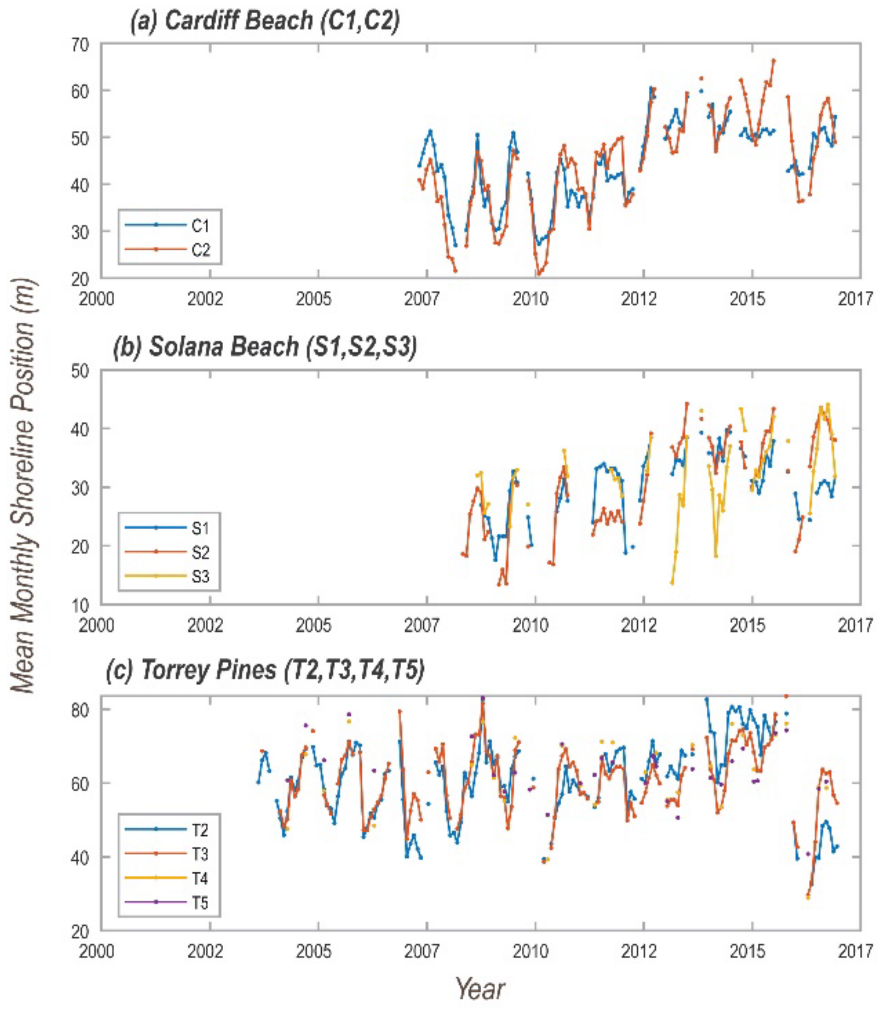

Field-surveyed shoreline positions for three beaches of the Scripps Institute of Oceanography study sites in San Diego County, California and used for comparative sites with our CoastSat measurements. (a) Cardiff Beach, sites C1 and C2, (b) Solana Beach, sites S1, S2 and S3, (c) Torrey Pines, sites T2, T3, T4 and T5.

Supplemental Fig. S11. Comparison of mean annual satellite-derived shorelines and field-surveyed shorelines.

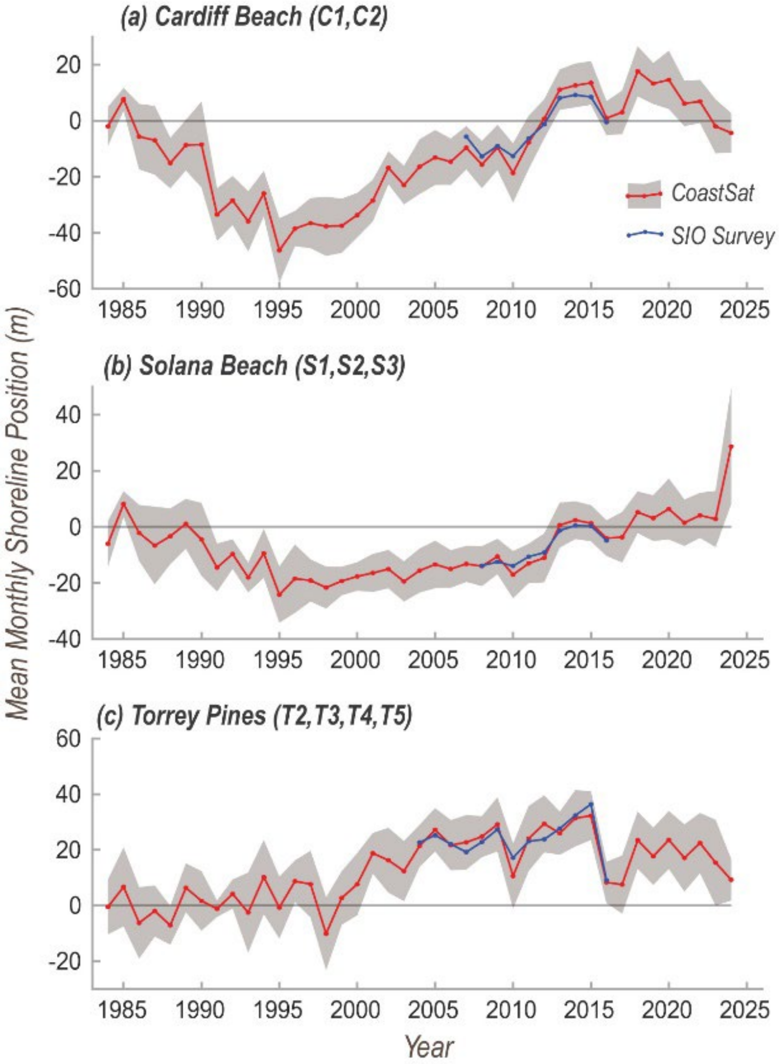

Time-series measurements of mean annual shoreline position from the Scripps Institute of Oceanography (SIO) field surveys (blue symbols) and the CoastSat satellite measurements (red symbols). The CoastSat data include the total 1-σ uncertainty (shading) for each measurement. (a) Cardiff Beach, sites C1 and C2, (b) Solana Beach, sites S1, S2 and S3, (c) Torrey Pines, sites T2, T3, T4 and T5.

Supplemental Fig. S12. Comparison of the mean annual satellite-derived shorelines and field-surveyed shorelines.

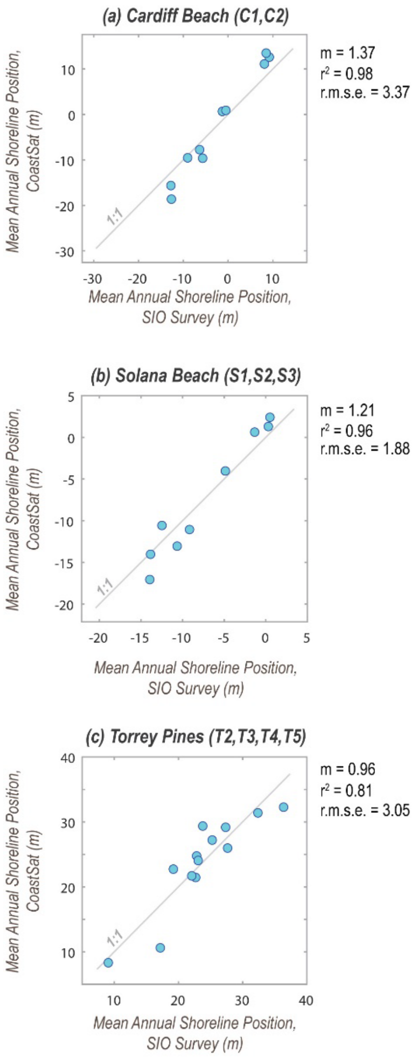

Comparisons of the measurements of mean annual shoreline position from the Scripps Institute of Oceanography (SIO) field surveys and the CoastSat satellite measurements. Lines represent the 1:1 line of perfect agreement. Linear egression statistics are included for each comparison, including the fitted slope (*m*), the correlation coefficient (r^2), and the root mean squared error (*r.m.s.e.*). (a) Cardiff Beach, sites C1 and C2, (b) Solana Beach, sites S1, S2 and S3, (c) Torrey Pines, sites T2, T3, T4 and T5.

Supplemental Fig. S13. Sediment transport and Peclet number for four southern California littoral cells.

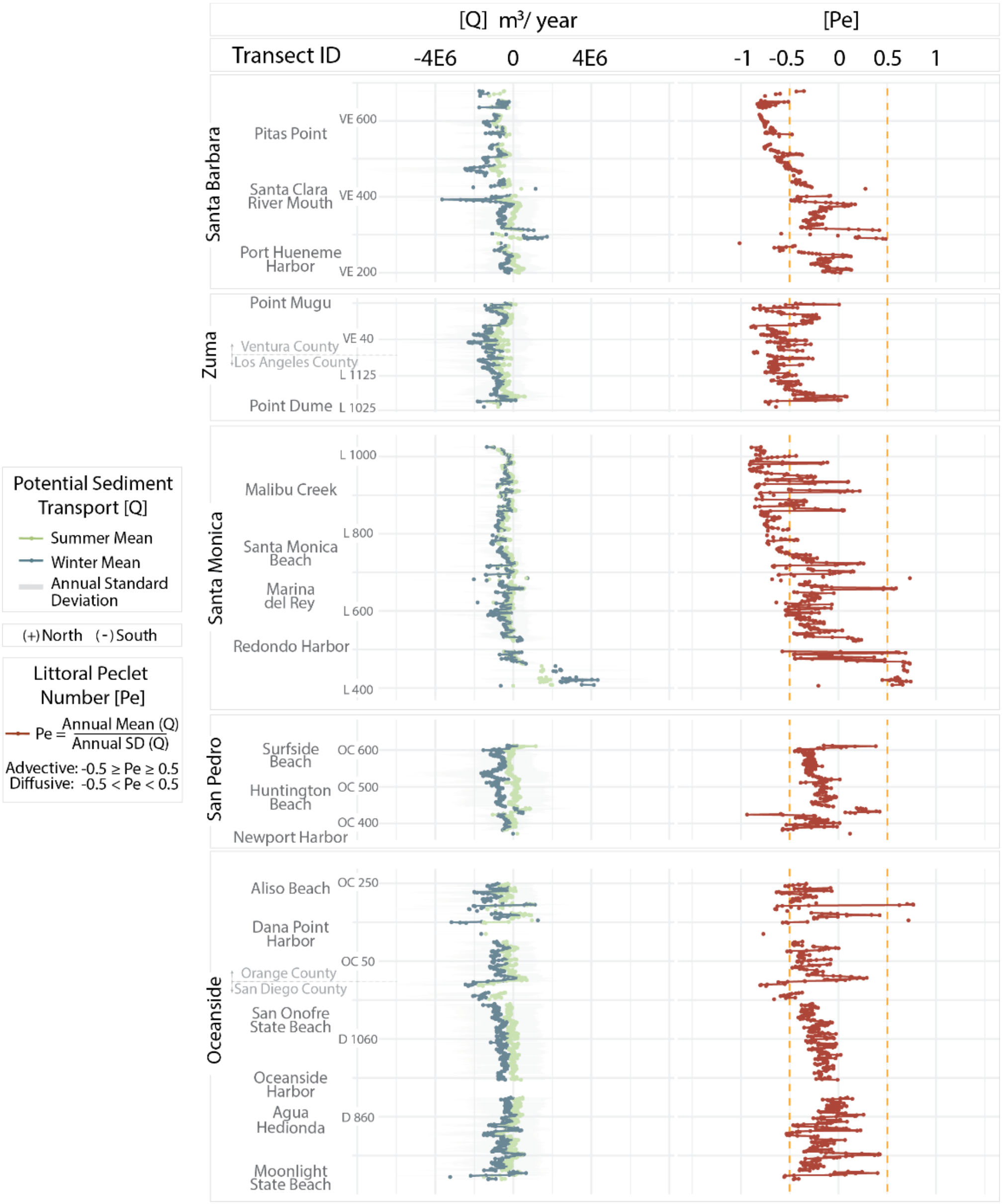

Potential sediment transport (Q) and Peclet number (Pe) results for five southern California littoral cells computed at 100-m longshore intervals. Hourly wave data and corresponding transect IDs from CDIP-MOP4 were obtained for a period of 24 years (2000-2024), for which transects are labeled by county (VE = Ventura, LA = Los Angeles, OC = Orange County, D = San

Diego) and the transect number in the longshore order (minimum value = most downcoast, maximum value = most upcoast). Additional features such as beaches, harbors, and county boundaries are included to support geographic perception. Seasonally averaged potential sediment transport for summer (April to September) and Winter (October to March) are shown with light and dark green, respectively. The Pe values are computed as the annual average Q over the annual average standard deviation in Q. Orange dashed lines are set at Pe values of -0.5 and 0.5, which designate approximate boundaries between longshore transport dominated by diffusion ($-0.5 < Pe < 0.5$) and advection ($|Pe| > 0.5$).

Supplemental Table S1. Sensitivity of linear trend in beach change to initial year of the regression.

Initial Year	Linear Trend (m^2/yr)	Percent of Original Trend
1984	56,392	100.0%
1985	54,978	97.5%
1986	56,465	100.1%
1987	55,831	99.0%
1988	56,157	99.6%
1989	54,974	97.5%
1990	57,123	101.3%
1991	62,589	111.0%
1992	59,143	104.9%
1993	55,310	98.1%

To evaluate the potential effect of the shoreline record duration on the computations of linear regression slope (i.e., linear trend), linear regression results are presented for the integrated southern California beach area data by changing the initial year included from the entire record (1984-2024). The range of computed trends varies by only -2.5% to 11% (i.e., 97.5% to 111% of the original 1984-2024 trend) with these different initial years, providing evidence that the initial start date has a minor effect on the trend results.

Supplemental Table S2. Beach nourishment volumes in southern California.

Littoral Cell	Locations	Total Volume (Mm^3/year) 1984 - 2024	Number of Projects
**Santa Barbara**	Ventura	0.34	36
	Channel Islands Maintenance Dredging	0.49	18
	Port Hueneme	0.14	12
	Total Annual Rate	**0.97**	66
**Zuma**	-	0	0
	Total Annual Rate	**0**	0
**Santa Monica**	Dockweiler State Beach	0.02	6
	Marina del Rey	0.061	9
	El Segundo	0.059	4
	Redondo Beach	0.027	8
	Total Annual Rate	**0.17**	27
**San Pedro**	San Pedro	0.004	1
	Long Beach	0.009	2
	Seal Beach	0.008	9
	Surfside-Sunset	0.14	4
	Huntington Beach	0.21	7
	Bolsa Chica Non-Federal Beach	0.013	3
	Newport Beach	0.064	11
	Total Annual Rate	**0.44**	37
**Oceanside**	Doheny Beach*	0.002	3
	Capistrano Beach*	0.002	2
	North Beach*	0.001	3
	San Clemente Pier*	0.004	2
	San Clemente	0.0003	2
	Oceanside	0.18	38
	Carlsbad	0.093	12
	Leucadia	0.003	1
	Total Annual Rate	**0.28**	63
* Calculation includes nourishment records supplementary to the ASBPA database			

Average beach nourishment rate (Mm^3/year) and total number of nourishment projects across littoral cells are calculated for the study duration (1984 - 2024) based on ASBPA Beach Nourishment Database[4] and additional data derived from local records[5–9].

References Cited

1. Cleveland, R. B., Cleveland, W. S., McRae, J. E. & Terpenning, I. STL: A seasonal-trend decomposition procedure based on loess. *Journal of Official Statistics* **6**, 3–73 (1990).
2. Warrick, J. A. & et al. Shoreline Seasonality of California's Beaches. *JGR Earth Surface* https://doi.org/10.1029/2024JF007836 doi:10.1029/2024JF007836.
3. Ludka, B. C. & and 15 others. Sixteen years of bathymetry and waves and San Diego beaches. *Scientific Data* **6**, (2019).
4. Elko, N. *et al.* A century of U.S. beach nourishment. *Ocean & Coastal Management* **199**, 105406 (2021).
5. San Clemente Sand Replenishment | City of San Clemente, CA. https://www.san-clemente.org/departments-services/planning-services/long-range-planning-projects/beach-restoration-project.
6. OC Parks | OC Parks. https://ocparks.com/ (2025).
7. Times, D. P. Harbor Dredging Project to Deposit Sand at Capo Beach. *Picket Fence Media* https://www.picketfencemedia.com/harbor-dredging-project-to-deposit-sand-at-capo-beach/article_071a1985-e411-56b5-981d-40d496bbe985.html (2016).
8. Parks, C. S. Major Sand Replenishment Project Underway at Doheny State Beach and Capistrano Beach Park. *California State Parks* https://www.parks.ca.gov/NewsRelease/1181.
9. Hrvacevic, Z. Sand deliveries complete in North Beach. *Dredging Today* https://www.dredgingtoday.com/2024/10/14/sand-deliveries-complete-in-north-beach/ (2024).
